# Smoking and nicotine use among Ukrainian migrants in Germany: a community-based survey of prevalence, vulnerabilities, and harm-reduction needs

**DOI:** 10.1192/j.eurpsy.2026.11634

**Published:** 2026-07-31

**Authors:** T. Pikirenia, K. Parfeniuk, U. Pikirenia, T. Jerzyński

**Affiliations:** 1 Knowledge Action Change, London, United Kingdom; 2 Association of Positive Ukrainians in Germany (PlusUkrDe e.V.), Berlin, Germany; 3 St. Anthony the Great Psychiatric Hospital in Frombork, Frombork; 4 R.B. Zajonc Institute for Social Studies, University of Warsaw, Warsaw, Poland

## Abstract

**Introduction:**

After Russia started a war on Ukraine, around 1.3 mln people were forced to migrate to Germany. Among them people with diverse health vulnerabilities, such as chronic infectious diseases and mental health issues. Being under enormous stress and having limited access to support, migrants can choose to use maladaptive coping mechanisms, practicing health-risk behaviors such as smoking.

**Objectives:**

To estimate the prevalence of nicotine use and predominant forms; describe prior harm reduction/cessation attempts and methods; explore additional vulnerabilities and need for future support.

**Methods:**

The questionnaire was developed in Google Forms and consisted of 11 questions. The survey was voluntary and anonymous; respondents could stop at any time without consequences; proceeding past the landing page and clicking “Next” after reading the information constituted informed consent; the EU’s GDPR was referenced and the data controller was indicated. The questionnaire was co-designed with the survey’s target group representatives from NGO “PlusUkrDe e.V.”, which took responsibility for the distribution of the survey among Ukrainian migrants in Germany in closed community groups. The ethics of the process and the truthfulness of the responses was ensured by the community nature of the research.

**Results:**

A total of 100 participants provided valid responses. Nearly all respondents (89%) reported moving to Germany from Ukraine due to the war that began in 2022. 62% of the respondents identified themselves as female, 35% – male, 3% – as non-binary. Respondents’ ages ranged from 18 to 74 years old, with the largest cluster between 25 and 45 years. Overall, 72% used nicotine/tobacco (28% did not). Most-used products were regular cigarettes – 48.6%; heated tobacco – 11.1%, e-cigarettes – 5.6%; pouches/snus were rare (1.4%). 87% of respondents identified themselves with one or more vulnerable groups. A considerable share of participants (37%) disclosed an official diagnosis of a mental health disorder (e.g., depression, PTSD, anxiety). 52% of respondents had attempted to reduce smoking harm or quit: 12% reported cutting down cigarettes, 19% had successfully switched to another product, and 21% had tried but not switched. 42% had not tried, and 6% were unaware of harm-reduction options. The most common quitting pathway was e-cigarettes with continued cigarette use (dual use; 37.1%); heated tobacco was used by 17.7%, and complete cessation was reported by 14.5%.

**Conclusions:**

The prevalence of nicotine use in the surveyed group is notably high. Almost half of them use the most harmful nicotine product. Combined with the high prevalence of mental health disorders and other reported vulnerabilities, smoking poses a significant health threat to forced Ukrainian migrants. They lack information on the ways to reduce harm and quit smoking with more probability of success.

**Disclosure of Interest:**

None Declared

